# Design, Synthesis and Anti-fibrosis Activity Study of N_1_-Substituted Phenylhydroquinolinone Derivatives

**DOI:** 10.3390/molecules17021373

**Published:** 2012-02-02

**Authors:** Ling Wu, Bin Liu, Qianbin Li, Jun Chen, Lijian Tao, Gaoyun Hu

**Affiliations:** 1 Department of Medicinal Chemistry, School of Pharmaceutical Sciences, Central South University, Changsha 410013, Hunan, China; Email: wl_nancy08@163.com (L.W.); qbli@csu.edu.cn (Q.L.); yxychenjun@csu.edu.cn (J.C.); 2 Division of Nephrology, Xiangya Hospital, Central South University, Changsha 410008, Hunan, China; Email: liubin_ooo@163.com (B.L.); taolj@csu.edu.cn (L.T.)

**Keywords:** N_1_-substituted phenylhydroquinolinones, synthesis, anti-fibrosis

## Abstract

Pirfenidone (5-methyl-1-phenyl-2(1H)-pyridone, PFD) is a small-molecule compound acting on multiple targets involved in pathological fibrogenesis and is effective to increase the survival of patients with fibrosis, such as idiopathic pulmonary fibrosis. However, PFD is not active enough, requiring a high daily dose. In this study, to keep the multiple target profiles, N_1_-substituted phenylhydroquinolinone derivatives, which retain the 1-phenyl-2(1*H*)-pyridone scaffold were designed and synthesized. The preliminary anti-fibrosis activities for all target compounds were evaluated on a NIH3T3 fibroblast cell line using MTT assay methods. Most compounds showed significant inhibition on NIH3T3 cell proliferation with a IC_50_ range of 0.09–26 mM, among which 5-hydroxy-1-(4'-bromophenyl)-5,6,7,8-tetrahydroquinolin-2(1*H*)-one (**6j**) displayed 13 times higher potency (IC_50_ = 0.3 mM) than that of AKF-PD (IC_50_ = 4.2 mM). These results suggest that N_1_-substituted phenylhydroquinolinone is a promising scaffold which can be applied for further investigation and for developing novel anti-fibrosis agents.

## 1. Introduction

Fibrosis is a pathological process in damaged tissues or organs where fibroblasts are activated to produce and deposit excess extracellular matrix (mainly composed of collagens). As more and more fibrous connective tissue is formed, the normal architecture is obliterated, resulting in scarring and dysfunction of the involved organs, such as liver and lung [1,2]. Currently, treatments for fibrotic diseases such as idiopathic pulmonary fibrosis [3], liver cirrhosis, systemic sclerosis, progressive kidney disease and cardiovascular fibrosis typically target the inflammatory response [4]. However, accumulating evidence indicates that the mechanisms driving fibrogenesis might be distinct from those regulating inflammation [5]. In fact, some studies have suggested that ongoing inflammation is favorable to reverse established and progressive fibrosis. Fibrogenesis is a complicated process during which mediators including cytokines (IL-13, IL-21, TGF-β1), angiogenic factors (VEGF), growth factors (PDGF) [6], chemokines (MCP-1, MIP-1β), peroxisome proliferator-activated receptors (PPARs) [7], acute phase proteins (SAP) [8], caspases, components of the rennin-angiotensin-aldosterone system (ANG II) [9] and p38 [10] have all been identified as important regulators and are being investigated as potential targets for the development of anti-fibrosis agents [11].

Therefore, the pathological mechanism of fibrosis is so complicated that none of the single-target agents have been marketed up to now, which has promoted the development of multiple-target anti-fibrosis drugs as represented by pirfenidone (5-methyl-1-phenyl-2-(*1H*)-pyridone, PFD). The mode of mechanism for PFD lies in interacting with multiple regulatory factors in the development of fibrosis. *In vitro*, PFD can inhibit p38 [10], heat shock protein 47 (HSP47) [12], TGF-β1, TNF-α [13], angiogenic factors (VEGF) and growth factors (PDGF) [14], which means it is more favorable to target multiple factors for the inhibition of fibrosis. *In vivo*, PFD inhibits the progression of fibrosis in animal models and is now used as anti-fibrosis therapy for patients with idiopathic pulmonary fibrosis (IPF) [15].

**Figure 1:**
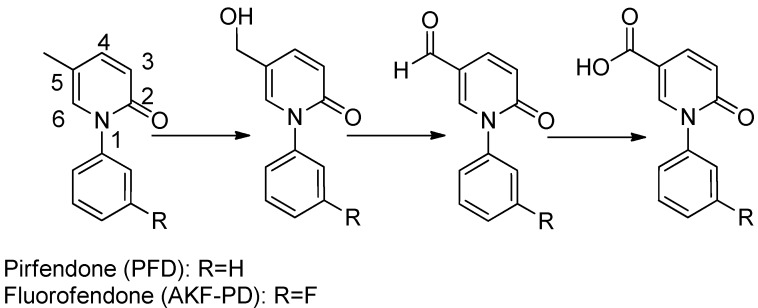
Chemical structure of PFD and AKF-PD and corresponding metabolism pathway.

However, during clinical application, PFD is not active enough due to its high daily dose, which is about 1,200 mg/day [15]. In addition, the oral LD_50_ of PFD in rat is as much as 1,121 mg/kg, which make PFD not beneficial for fibrosis patients needing long-term treatment. Previously, our group discovered fluorofenidone (AKF-PD) [16], which showed lower toxicity but still equivalent activity to PFD. The metabolism pathway of PFD and AKF-PD was also studied and demonstrated in further studies on NIH3T3 cell line [17]. As shown in Figure 1, the 5-CH_3_ was oxidized rapidly in turn into active hydroxyl group and aldehyde metabolites, and finally to inactive carboxylic acid, which is the main reasons for the lower activity of PFD.

**Figure 2:**
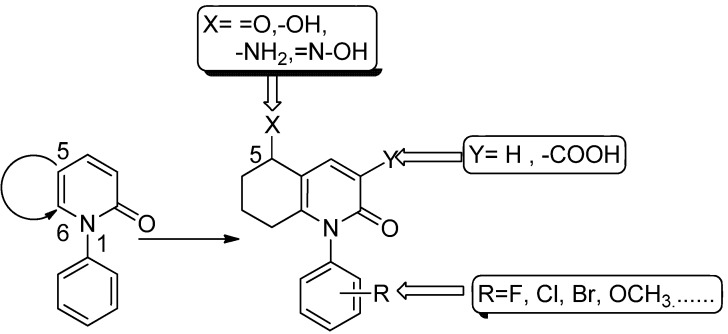
Design strategies for N_1_-substituted phenylhydroquinolinone derivatives.

Herein, to keep the multiple-target anti-fibrosis activity features of PFD and to prevent the rapid metabolism of 5-CH_3_, the C-5 and C-6 of PFD were cyclized to form an N_1_-substituted phenylhydroquinolinone scaffold (Figure 2). Additionally, hydrogen bond donors or acceptors at the C-5 of hydroquinolinones and different substituents on N-1 phenyl group were introduced. To evaluate the structure-activity relationship for all target compounds, the *in vitro* inhibitory activity was measured on NIH3T3 cell lines using MTT methods.

## 2. Results and Discussion

### 2.1. Chemistry

**Scheme 1:**
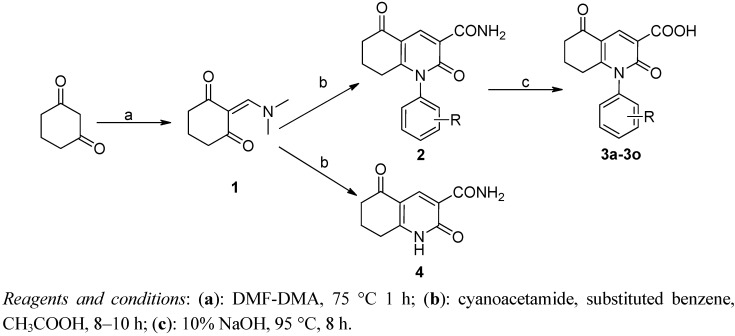
The chemical synthesis of **3**.

Synthesis of the target compounds **3** and analogs began with the synthesis of intermediates **1**. As shown in Scheme 1, condensation of 1,3-cyclohexanedione and DMF in DMA afforded **1**, which can yield different major compounds **2** or **4** when the concentration of compound **1** was 0.4 M or 2 M, respectively [18]. Hydrolysis of amide group in compound **2** under basic conditions for various amounts of time afforded 3-carboxylic acid analogs **3a**–**3o** in yields ranging from 60–90%. 

**Scheme 2:**
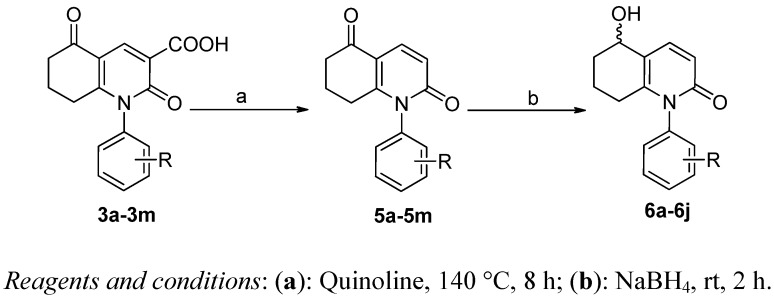
The synthesis of **5** and **6**.

**Scheme 3:**
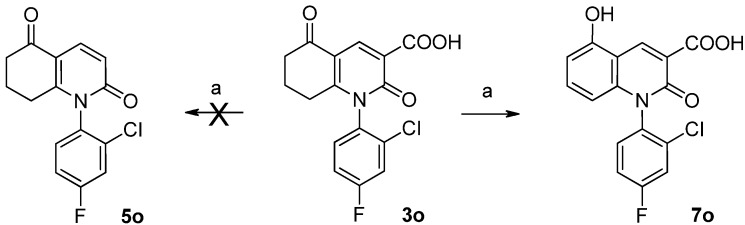
The conversion of compound **3o** to **7** instead of **5o**.

To gain further insight into the different role of hydrogen bond acceptors or donors on C-5 of the hydroquinolinones, compounds **6** and **5** were synthesized efficiently following the procedures shown in Scheme 2. 3-Carboxylic acid analogs **3** were decarboxylated in the presence of hot quinoline to afford **4**, which can be easily reduced to its hydroxyl derivative **5** by NaBH_4_ [19]. Interestingly, during the preparation of compound **5o** from **3o**, only the aromatized product **7** was obtained, instead of the expected decarboxylation product **5o** (Scheme 3). A possible reason for this may be the presence of two strong electron-withdrawing groups (2-Cl and 4-F), which can induce the dehydrogenation and aromatization of the cyclohex-2-enone part.

**Scheme 4:**
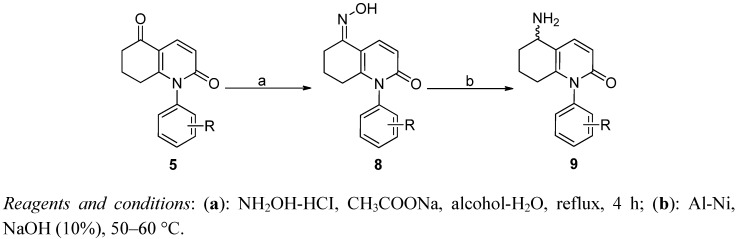
The synthesis of **8**.

To study the effect of different hydrogen bond donors at C-5 for compounds **5**, the carbonyl groups of compounds **5** were easily converted in a mixture of alcohol and water in the presence of NH_2_OH to its oxime derivatives **8** (Scheme 4), which can be reduced to primary amine analogs **9** by the catalyst Al-Ni in 10% NaOH [20].

### 2.2. Biological Assay

The anti-fibrosis activity of the target compounds **3**–**9** was evaluated on the NIH3T3 cell line using MTT methods with AKF-PD as control. All the inhibition results are summarized in Table 1. 

**Table 1:** The *in vitro* MTT assay results on NIH3T3 cell line for compound **3** and **5–9**.

No.	R	IC_50_ (mM)	No.	R	IC_50_ (mM)	No.	R	IC_50_ (mM)
**3a**	H	1	**5a**	H	5.4	**6a**	H	6.5
**3b**	o-Cl	20	**5b**	o-Cl	3.5	**6d**	p-Cl	1.5
**3c**	m-Cl	>1,000	**5c**	m-Cl	4.1	**6h**	o-F	1
**3d**	p-Cl	2	**5d**	p-Cl	2.8	**6i**	p-F	2.7
**3e**	o-CH_3_	>1,000	**5e**	o-CH_3_	3.5	**6j**	p-Br	0.3
**3f**	m-CH_3_	>1,000	**5f**	m-CH_3_	4.0	**6k**	p-OCH_3_	3.4
**3g**	p-CH_3_	18.7	**5g**	p-CH_3_	2.4	**6l**	2,4-di-Cl	1
**3h**	o-F	>1,000	**5h**	o-F	2.2	**6m**	2,4-di-CH_3_	1.7
**3i**	p-F	2.6	**5i**	p-F	4	**7o**	2-Cl, 4-F	0.09
**3j**	p-Br	4.2	**5j**	p-Br	2.2	**8i**	p-F	3.9
**3k**	p-OCH_3_	>1,000	**5k**	p-OCH_3_	2.5	**8k**	p-OCH_3_	83
**3l**	2,4-di-Cl	25.6	**5l**	2,4-di-Cl	6.2	**9a**	H	2.1
**3m**	2,4-di-CH_3_	15.6	**5m**	2,4-di-CH_3_	3.7	**-**	-	-
**3n**	3,4-di-Cl	0.3	-	-	-	-	-	-
**3o**	2-Cl, 4-F	2.7	-	-	-	AKF-PD		4.2

Bioassay results indicated that the inhibitory effect on NIH3T3 cell proliferation varied markedly for different series, with the IC_50_ values ranging from 0.09 mM > 1,000 mM. The existence of a 3-COOH group (series **3**) is less favorable for the anti-fibrosis activity compared to that of series **5** and **6**, which can be represented by compounds with *o*-F (**h**), *p*-Br (**j**), 2,4-di-Cl (**l**) and 2,4-di-CH_3_ (**m**) substituents on the N-1 phenyl group. Additionally, series **6** is more favorable than series **5** after the reduction of carbonyl to a hydroxyl group, 5-hydroxy-1-(4'-bromophenyl)-5,6,7,8-tetrahydroquinolin-2(1*H*)-one (**6j**) displayed 13 times higher potency than that of AKF-PD (IC_50_ values of 0.3 mM and 4.2 mM, respectively), which suggested the critical role of a hydrogen bond donor on C-5 of the hydroquinolinones. This activity requirement can also be demonstrated by the potency of compound **9a***vs.***6a**, in which the hydroxyl group is replaced by a free amino group on the C-5 position.

As for compounds in series **3**, the location of substituents on the N-1 phenyl group is more important for the anti-fibrosis activity than the type of substituents. Compounds with *para*-substitutents on the phenyl group are more potent than those with *ortho*- or *meta*-substitutents. Certain compounds, such as **3i** and **3j** displayed higher inhibitory potency than that of **3c**, **3e**, **3f** or **3h** with the IC_50_ value of 2.6 mM, 4.2 mM and >1,000 mM, respectively. In addition, compound **3n** (3,4-di-Cl substituent, IC_50_ = 0.3 mM) displayed more remarkable potency than compound **3l** (2,4-di-Cl substituent, IC_50_ = 25.6 mM).

As shown in Table 1, no obvious difference on the activity for location or type of substituents on the N-1 phenyl group can be observed for series **5** and **6**, both of which displayed higher potency. What’ s most interesting is that the aromatized compound 1-(2-chloro-4-fluorophenyl)-5-hydroxy-2-oxo-1,2-dihydroquinoline-3-carboxylic acid (**7o**) exhibited 46 times higher potency than that of AKF-PD (IC_50_ values of 0.09 mM and 4.2 mM, respectively), which is now applied for further modification. 

## 3. Experimental

### 3.1. General

All chemicals and solvents were from commercial sources and used without further treatment unless specified otherwise. The purity of all compounds was examined by high performance liquid chromatography (ODS column, 250 mm × 4.6 mm, 5 μm particle; mobile phase: acetonitrile in water; flow rate: 1.0 mL/min at 25 °C). ^1^H-NMR and ^13^C-NMR spectra were obtained using Me_4_Si as an internal standard on Bruker 400 or Varian 300 NMR instruments. Mass spectra were recorded on a Qstar LC/MS instrument. Chromatography was performed with commercial silica gel (300–400 mesh).

#### 3.1.1. *Preparation of 2-((Dimethylamino) methylene)cyclohexane-1,3-dione* (**1**)

To 1,3-cyclohexandione (50 g) was added DMF-DMA (140 mL) with stirring. The resulting solution was heated to 75 °C for one hour. Solvent was removed and the residue was recrystallized from EtOAc to yield slight yellow crystals. Yield: 90%, mp: 117–118 °C.

#### 3.1.2. General Procedure for the Preparation of **2**

*1-Phenyl-1,2,5,6,7,8-hexahydro-2,5-dioxo-3-quinolinecarboxylic* *acid* (**2a**). Compound **1** (10 g) was dissolved in the mixture of 2-propanol (70 mL) and piperidine (0.1 g, 0.11 mmol) was added to the reaction. The mixture was stirred at room temperature for 5 h and to the mixture was added aniline (8.0 g) in acetic acid (40 mL). After 3 h, crystallization of a yellow solid occurred, which was filtered and recrystallized from methanol to yield a white solid. Yield: 61%, mp: >300.0 °C. MS *m/z*: 283 (M^+^). ^1^H-NMR (400 MHz, DMSO-*d_6_*): δ 2.068–2.171 (m, 2H), 2.569–2.591 (m, 2H), 7.253–7.278 (m, 2H, Ar-H), 7.591–7.686 (m, 3H, Ar-H), 9.182 (s, 1H), 13.227 (s, 1H). ^13^C-NMR (100 MHz, DMSO-*d_6_*): δ 21.088, 29.514, 36.556, 116.537, 116.730, 127.243 (2C), 130.698 (2C), 130.819, 136.006, 143.862, 161.063, 164.239, 165.456, 192.657. 

#### 3.1.3. General Procedure for the Preparation of **3**

*1-Phenyl-1,2,5,6,7,8-hexahydro-2,5-dioxo-3-quinolinecarboxylic* *acid* (**3a**). To **2a** (8.0 g) was added NaOH (10%, 50 mL) with stirring. The resulting solution was heated to 95 °C for 8 h and allowed to cool to room temperature. The pH of the solution was adjusted to 2 with diluted HCl, and crystallization of a yellow solid occurred, which was filtered and recrystallized in methanol to yield a white solid. Yield: 85%, HPLC purity: 99.4%. mp: 259.8–260.6 °C. MS *m/z*: 283 (M^+^). ^1^H-NMR (400 MHz, DMSO-*d_6_*): δ 1.985–2.170 (m, 2H), 2.421–2.441 (m, 2H), 2.462–2.540 (m, 2H), 6.569–6.602 (d, 1H, *J* = 9.9 Hz), 7.197–7.262 (m, 2H, Ar-H), 7.480–7.587 (m, 3H, Ar-H), 8.049–8.081 (d, 1H, *J* = 9.6 Hz). ^13^C-NMR (100 MHz, DMSO-*d_6_*): δ 21.472, 28.924, 36.470, 114.684, 119.004, 127.771 (2C), 129.449, 130.133 (2C), 136.950, 137.491, 157.133, 163.212, 194.063. 

*1-(2'-Chlorophenyl)-1,2,5,6,7,8-hexahydro-2,5-dioxo-3-quinolinecarboxylic* *acid* (**3b**). Yield: 87%, HPLC purity: 99.1%, mp: 247.3–248.1 °C, MS *m/z*: 317 (M^+^). ^1^H-NMR (400 MHz, DMSO-*d_6_*): δ 2.101–2.581 (m, 2H), 2.606–2.692 (m, 2H), 2.498–2.529 (m, 2H), 7.4263 (m, 1H, Ar-H), 7.531–7.675 (m, 2H, Ar-H), 7.682–7.707 (m, 1H, Ar-H), 9.208 (s, 1H), 13.016 (s, 1H). ^13^C-NMR (100 MHz, DMSO-*d_6_*): δ 20.759, 28.372, 36.161, 114.577, 117.385, 129.316, 130.262, 130.728, 130.895, 132.017, 134.374, 141.705, 161.373, 162.540, 164.531, 193.551. 

*1-(3'-Chlorophenyl)-1,2,5,6,7,8-hexahydro-2,5-dioxo-3-quinolinecarboxylic* *acid* (**3c**). Yield: 91%, HPLC purity: 99.2%, mp: 247.3–248.1 °C, MS *m/z*: 317 (M^+^). ^1^H-NMR (400 MHz, DMSO-*d_6_*): δ 1.965–1.996(m, 2H), 2.498–2.529 (m, 4H), 7.444 (s, 1H, Ar-H), 7.451–7.667 (m, 3H, Ar-H), 8.686 (s, 1H), 13.222 (s, 1H).^13^C-NMR (100 MHz, DMSO-*d_6_*): δ 20.736, 29.272, 36.207, 114.829, 116.645, 127.089, 128.286, 130.133, 131.780, 134.229, 138.150, 141.477, 163.036, 163.119, 164.622, 193.772.

*1-(4'-Chlorophenyl)-1,2,5,6,7,8-hexahydro-2,5-dioxo-3-quinolinecarboxylic* *acid* (**3d**). Yield: 88%, HPLC purity: 99.0%, mp: 244.2–244.9 °C, MS *m/z*: 317 (M^+^). ^1^H-NMR (300 MHz, DMSO-*d_6_*): δ 2.085–2.171 (m, 2H), 2.286–2.374 (m, 2 H), 2.572–2.641 (m, 2H), 7.215–7.263 (m, 2H, Ar-H), 7.610–7.638 (m, 2H, Ar-H), 9.154 (s, 1H), 13.034 (s, 1H). ^13^C-NMR (100 MHz, DMSO-*d_6_*): δ 20.883, 29.374, 36.310, 116.456, 116.569, 128.632 (2C), 130.929 (2C), 134.171, 136.804, 143.762, 160.726, 16 3.861, 165.127, 192.295. 

*1-(2'-Methylphenyl)-1,2,5,6,7,8-hexahydro-2,5-dioxo-3-quinolinecarboxylic* *acid* (**3e**). Yield: 91%, HPLC purity: 99.0%, mp: 237.0–238.2 °C, MS *m/z*: 297 (M^+^). ^1^H-NMR (300-MHz, DMSO-*d_6_*): δ 2.055–2.199 (m, 5H), 2.316–2.415 (m, 1H), 2.531–2.699 (m, 3H, CH_3_), 7.132–7.157 (m, 1H, Ar-H), 7.263–7.538 (m, 3H, Ar-H), 9.202 (s, 1H), 13.232 (s, 1H). ^13^C-NMR (100 M Hz, DMSO-*d_6_*): δ 17.005, 20.705, 28.586, 36.169, 115.272, 116.553, 127.829, 127.913, 130.209, 131.643, 134.870, 135.969, 141.530, 162.769, 162.837, 164.615, 193.787.

*1-(3'-Methylphenyl)-1,2,5,6,7,8-hexahydro-2,5-dioxo-3-quinolinecarboxylic* *acid* (**3f**). Yield: 87%, HPLC purity: 99.2%, mp: 219.2–219.9 °C, MS *m/z*: 297 (M^+^). ^1^H-NMR (400 MHz, DMSO-*d_6_*): δ 2.407 (m, 3H, CH_3_), 2.254 (m, 6H), 7.220–7.504 (m, 4H, Ar-H), 8.705 (s, 1H), 13.381 (s, 1H). ^13^C-NMR (100 MHz, DMSO-*d_6_*): δ 20.667, 21.087, 29.333, 36.207, 115.104, 116.149, 127.638 (2C), 130.636 (2C), 134.252, 139.638, 141.355, 163.539, 163.837, 164.683, 193.848.

*1-(4'-Methylphenyl)-1,2,5,6,7,8-hexahydro-2,5-dioxo-3-quinolinecarboxylic* *acid* (**3g**). Yield: 86%, HPLC purity: 99.1%, mp: 244.2–244.9 °C, MS *m/z*: 297 (M^+^). ^1^H-NMR (400 MHz, DMSO-*d_6_*): δ 2.387–2.503 (m, 3H, CH_3_), 2.503–2.520 (m, 6H), 7.294–7.426 (m, 4H, Ar-H), 8.704 (s, 1H), 13.403 (s, 1H). ^13^C-NMR (100 MHz, DMSO-*d_6_*): δ 20.667, 21.087, 29.333, 36.2 07, 115.104, 116.149, 127.638 (2C), 130.636 (2C), 134.252, 139.638, 141.355, 163.837, 16 4.683, 193.848. 

*1-(2'-Fluorophenyl)-1,2,5,6,7,8-hexahydro-2,5-dioxo-3-quinolinecarboxylic* *acid* (**3h**). Yield: 87%, HPLC purity: 99.1%, mp: 210.4–212.2 °C, MS *m/z*: 301 (M^+^). ^1^H-NMR (400 MHz, DMSO-*d_6_*): δ 1.986–2.01 6m, 2H), 2.457–2.544 (m, 4H), 7.462–7.601 (m, 4H, Ar-H), 8.673 (s, 1H), 13.099 (s, 1H). ^13^C-NMR (100 MHz, DMSO-*d_6_*): δ 20.636, 28.555, 36.077, 114.715, 116.988, 117.179, 117.248, 126.051, 130.239, 132.528, 132.604, 141.644, 161.479, 162.799, 164.508, 193.536. 

*1-(4'-Fluorophenyl)-1,2,5,6,7,8-hexahydro-2,5-dioxo-3-quinolinecarboxylic* *acid* (**3i**). Yield: 89%, HPLC purity: 99.1%, mp: 260.4–261.3 °C, MS *m/z*: 301 (M^+^). ^1^H-NMR (400 MHz, DMSO-*d_6_*): δ 2.059–2.170 (m, 2H), 2.571–2.641 (m, 4H), 7.147–7.263 (m, 4H, Ar-H), 8.698 (s, 1H), 13.298 (s, 1H).^13^C-NMR (100 MHz, DMSO-*d_6_*): δ 20.422, 29.035, 35.918, 114.652, 116.243, 122.999, 130.074 (2C), 132.931 (2C), 135.934, 141.172, 162.83, 164.356, 193.500.

*1-(4'-Bromophenyl)-1,2,5,6,7,8-hexahydro-2,5-dioxo-3-quinolinecarboxylic* *acid* (**3j**). Yield: 86%, HPLC purity: 99.1%, mp: 256.0–257.3 °C, MS *m/z*: 364 (M^+^).^1^H-NMR (300 MHz, DMSO-*d_6_*): δ 2.059–2.170 (m, 2H), 2.571–2.641 (m, 4H), 7.147–7.263(m, 2H, Ar-H), 7.768–7.797 (m, 2H, Ar-H), 9.161 (s, 1H), 13.232 (s, 1H). ^13^C-NMR (100 MHz, DMSO-*d_6_*): δ 20.422, 29.035, 35.918, 114.652, 116.243, 122.999, 130.074 (2C), 132.931 (2C), 135.934, 141.172, 162.83, 164.356, 193.500.

*1-(4'-Methoxyphenyl)-1,2,5,6,7,8-hexahydro-2,5-dioxo-3-quinolinecarboxylic* *acid* (**3k**). Yield: 90%, HPLC purity: 99.0%, mp: 254.5–255.6 °C, MS *m/z*: 313 (M^+^). ^1^H-NMR (300 MHz, DMSO-*d_6_*): δ 2.066–2.170 (m, 2H), 2.585–2.637 (m, 4H), 3.897 (s, 3H, OH), 7.097–7.262 (m, 4H, Ar-H), 9.173 (s, 1H), 13.271 (s, 1H). ^13^C-NMR (100 MHz, DMSO-*d_6_*): δ 20.659, 2.349, 36.192, 55.798, 115.127, 115.256 (2C), 116.019, 129.057 (2C), 129.209, 141.316, 160.060, 163.875, 164.104, 164.668, 193.841.

*1-(2',4’-Dichlorophenyl)-1,2,5,6,7,8-hexahydro-2,5-dioxo-3-quinoline* *carboxylicacid* (**3l**). Yield: 91%, HPLC purity: 99.0%, mp: 263.0–266.3 °C, MS *m/z*: 353 (M^+^). ^1^H-NMR (400 MHz, MSO-*d_6_*): δ 1.956–2.040 (m, 2H), 2.472–2.631 (m, 4H), 7.147–7.263 (m, 1H, Ar-H), 7.727–7.754 (m, 1H, Ar-H), 8.020–8.025(m, 1H, Ar-H), 8.669 (s, 1H), 13.040 (s, 1H). ^13^C-NMR (100 MHz, DMSO-*d_6_*): δ 20.443, 28.011, 35.838, 114.171, 129.215, 130.092, 131.343, 132.014, 133.243, 135.432, 141.405, 160.615, 162.125, 164.185, 193.174. 

*1-(2',4’-Diethylphenyl)-1,2,5,6,7,8-hexahydro-2,5-dioxo-3-quinoline* *carboxylicacid* (**3m**). Yield: 87%, HPLC purity: 99.0%, mp: 240.5–241.1 °C, MS *m/z*: 311 (M^+^). ^1^H-NMR (300 MHz, DMSO-*d_6_*): δ 2.049 (s, 3H, CH_3_), 2.076–2.191 (m, 2H), 2.431 (s, 3H, CH_3_), 2.335–2.397(m, 1H), 2.527–2.708 (m, 3H), 6.995–7.021 (m, 1H, Ar-H), 7.233–7.274 (m, 1H, Ar-H), 9.202(s, 1H), 13.316 (s, 1H). ^13^C-NMR (100 MHz, DMSO-*d_6_*): δ 17.432, 21.188, 21.447, 28.945, 36.656, 116.655, 116.768, 126.742, 129.116, 132.971, 134.163, 141.232, 143.916, 161.133, 164.331, 165.070, 192.673. 

*1-(3',4'-Dichlorophenyl)-1,2,5,6,7,8-hexahydro-2,5-dioxo-3-quinoline* *carboxylicacid* (**3n**). Yield: 86%, HPLC purity: 99.1%, mp: 224.0–225.2 °C, MS *m/z*: 353 (M^+^). ^1^H-NMR (400 MHz, DMSO-*d_6_*): δ 1.956–2.006 (m, 2H), 2.468–3.352 (m, 4H), 7.505–7.527 (m, H, Ar-H), 7.890–7.931 (m, 2H, Ar-H), 8.671 (s, H), 13153 (s, 1H). ^13^C-NMR (100 MHz, DMSO-*d_6_*): δ 20.913, 29.423, 36.379, 114.917, 117.029, 129.020, 130.706, 132.245, 132.645, 133.148, 136.856, 141.684, 162.907, 163.218, 164.747, 193.874. 

*1-(2'-Chloro-4'-ﬂuorophenyl)-1,2,5,6,7,8-hexahydro-2,5-dioxo-3-quinoline* *carboxylicacid* (**3o**). Yield: 86%, HPLC purity: 99.0%, mp: 337.8–338.7 °C, MS *m/z*: 336 (M^+^). ^1^H-NMR (400 MHz, DMSO-*d_6_*): δ 1.956–1.988 (m, 2H), 2.469–2.506(m, 4H), 7.661–7.853 (m, 3H), 8.435–8.444 (1H), 8.806 (COOH).^13^C-NMR (100 MHz, DMSO-*d_6_*): δ 20.512, 28.812, 35.922, 113.927, 117.764, 118.984, 120.533, 129.123, 130.512, 134.028, 140.002, 156.198, 158.677, 161.492, 162.232, 163.521, 193.579. 

#### 3.1.4. General Procedure for the Preparation of **5**

*1-Phenyl-7,8-dihydro-2,5(1*H*,6*H*)-quinolinedione* (**5a**). To dried quinolone (20 mL) and **3a** (5.0 g) copper (0.1 g) was added. The mixture was stirred and heated to 140–150 °C and refluxed for 8 h. After the suspension was cooled in the air to room temperature, **5a** was obtained upon column chromatography (EtOAc/petroleum ether) to yield a white solid. Yield: 53%, HPLC purity: 99.1%, mp: 138.4–139.3 °C, MS *m/z*: 239 (M^+^). ^1^H-NMR (400 MHz, DMSO-*d_6_*): δ 1.985–2.170 (m, 2H), 2.421–2.441 (m, 2H), 2.462–2.540 (m, 2H), 6.569–6.602 (d, 1H, *J* = 9.9 Hz), 7.197–7.262 (m, 2H, Ar-H), 7.480–7.587 (m, 3H, Ar-H), 8.049–8.081 (d, 1H, *J* = 9.6 Hz). ^13^C-NMR (100 MHz, DMSO-*d_6_*): δ 21.472, 28.924, 36.470, 114.684, 119.004, 127.771 (2C), 129.449, 130.133 (2C), 136.950, 137.491, 157.133, 163.212, 194.063.

*1-(2'-Chlorophenyl)-7,8-dihydro-2,5(1*H*,6*H*)-quinolinedione* (**5b**). Yield: 36%, HPLC purity: 99.2%, mp: 161.2–161.9 °C, MS *m/z*: 273 (M^+^). ^1^H-NMR (300 MHz, DMSO-*d_6_*): δ 2.019–2.104 (m, 2H), 2.342–2.508 (m, 2H), 2.517–2.618 (m, 2H), 6.575–6.607 (d, 1H, *J* = 8.8 Hz), 7.265–7.306 (m, 1H, Ar-H), 7.444–7.505 (m, 2H, Ar-H), 7.444–7.505 (m, 2H, Ar-H), 7.587–7.643 (m, 1H, Ar-H), 8.066–8.098 (d, 1 H, *J=* 9.6 Hz). ^13^C-NMR (100 MHz, DMSO-*d_6_*): δ 21.193, 27.981, 36.222, 114.059, 118.255, 129.110, 130.583, 130.644, 131.330, 131.468, 135.267, 137.258, 158.275, 161.578, 194.008. 

*1-(3'-Chlorophenyl)-7,8-dihydro-2,5(1*H*,6*H*)-quinolinedione* (**5c**). Yield: 36%, HPLC purity: 99.3%, mp: 184.3–185.4 °C, MS *m/z*: 273 (M^+^). ^1^H-NMR (400 MHz, DMSO-*d_6_*): δ 1.932 (m, 2H), 2.404–2.437 (m, 4H), 6.463–6.487 (d, 1H, *J* = 9.6 Hz), 7.339–7.366 (s, 1H, Ar-H), 7.564 (m, 1H, Ar-H), 7.564–7.608 (m, 2H, Ar-H), 7.925–7.948 (d, 1H, *J* = 9.6 Hz). ^13^C-NMR (100 MHz, DMSO-*d_6_*): δ 21.125, 28.692, 36.192, 113.876, 118.010, 127.470, 128.675, 129.438, 131.460, 134.000, 136.922, 139.112, 158.557, 162.257, 194.062. 

*1-(4'-Chlorophenyl)-7,8-dihydro-2,5(1*H*,6*H*)-quinolinedione* (**5d**). Yield: 41%, HPLC purity: 99.6%, mp: 144.0–144.5 °C, MS *m/z*: 273 (M^+^). ^1^H-NMR (300 MHz, DMSO-*d_6_*): δ 2.007–2.069 (m, 2H), 2.424–2.533 (m, 4H), 6.558–6.582 (d, 1H, *J* = 9.6 Hz), 7.148–7.260 (m, 2H, Ar-H), 7.519–7.539 (m, 2H, Ar-H), 8.041–8.065 (d, 1 H, *J* = 9.6 Hz). ^13^C-NMR (100 MHz, DMSO-*d_6_*): δ 21.581, 29.100, 36.560, 114.997, 119.174, 129.389 (2C), 130.567 (2C), 135.743, 137.304, 156.917, 163.224, 194.068. 

*1-(2'-Methylphenyl)-7,8-dihydro-2,5(1*H*,6*H*)-quinolinedione* (**5e**). Yield: 40%, HPLC purity: 99.1%, mp: 144.1–144.9 °C, MS *m/z*: 253 (M^+^). ^1^H-NMR (300 MHz, DMSO-*d_6_*): δ 1.974–2.094 (m, 5H), 2.167–2.264 (m, 1H), 2.444–2.549 (m, 3H, CH_3_), 6.582–6.614 (d, 1H, *J=* 9.6 Hz), 7.088–7.111 (m, 1H, Ar-H), 7.265–7.417 (m, 3H, Ar-H), 8.065–8.098 (d, 1H, *J* = 9.9 Hz). ^13^C-NMR (100 MHz, DMSO-*d_6_*): δ 17.371, 21.257, 28.257, 36.476, 114.747, 119.009, 127.501, 127.741, 129.707, 131.622, 134.972, 136.593, 136.933, 157.006, 162.603, 194.061. 

*1-(3'-Methylphenyl)-7,8-dihydro-2,5(1*H*,6*H*)-quinolinedione* (**5f**). Yield: 39%, HPLC purity: 99.1%, mp: 122.6–123.0 °C. MS *m/z*: 253 (M^+^).^1^H-NMR (300 MHz, DMSO-*d_6_*): δ 1.983–2.068 (m, 2H), 2.425–2.475 (m, 3H, CH_3_), 2.491–2.536 (m, 4 H), 6.563–6.595 (d, 1H, *J* = 9.6 Hz), 6.988–7.014 (m, 2H, Ar-H), 7.263–7.318 (m, 1H, Ar-H), 7.412–7.463 (m, 1H, Ar-H), 8.038–8.070 (d, 1H, *J* = 9.6 Hz).^13^C-NMR (100 MHz, DMSO-*d_6_*): δ 21.653, 21.703, 29.115, 36.720, 114 .868, 119.186, 124.869, 128.404, 130.511, 137.123, 137.618, 140.584, 157.482, 163.527, 194.360 

*1-(4'-Methyphenyl)-7,8-dihydro-2,5(1*H*,6*H*)-quinolinedione* (**5g**). Yield: 40%, HPLC purity: 99.0%, mp: 196.5–197.2 °C, MS *m/z*: 253 (M^+^). ^1^H-NMR (300 MHz, DMSO-*d_6_*): δ 1.980–2.065 (m, 2H), 2.437–2.478 (m, 3H, CH_3_), 2.491–2.535 (m, 4H), 6.563–6.595 (d, 1H, *J=* 9.6 Hz), 7.068–7.095 (m, 2H, Ar-H), 7.263–7.366 (m, 2H, Ar-H), 8.036–8.068 (d, 1H, *J* = 9.6 Hz).^13^C-NMR (100 MHz, DMSO-*d_6_*): δ 21.467, 21.076, 36.627, 114.815, 119.092, 127.551 (2C), 130.951 (2C), 134.934, 137.021, 139.724, 157.520, 163.546, 194.309. 

*1-(2'-Fluorophenyl)-7,8-dihydro-2,5(1*H*,*6H*)-quinolinedione* (**5h**). Yield: 41%, HPLC purity: 99.0%, mp: 207.8–208.7 °C, MS *m/z*: 257 (M^+^). ^1^H-NMR (400 MHz, DMSO-*d_6_*): δ 1.913–1.992 (m, 2H), 2.269–2.618 (m, 4H), 6.499–6.523 (d, 1H, *J* = 9.6 Hz), 7.349–7.632 (m, 4H, Ar-H), 7.955–7.979 (d, 1H, *J* = 9.9 Hz).^13^C-NMR (100 MHz, DMSO-*d_6_*): δ 21.102, 28.097, 36.169, 114.219, 116.866 , 117.988, 24.907, 125.860, 130.651, 131.948, 137.321, 155.979, 158.443, 161.716, 194.047.

*1-(4'-Fluorophenyl)-7,8-dihydro-2,5(1*H*,6*H*)-quinolinedione* (**5i**). Yield: 42%, HPLC purity: 99.0%, mp: 177.2–178.2 °C, MS *m/z*: 257 (M^+^). ^1^H-NMR (400 MHz, DMSO-*d_6_*): δ 1.911–1.943 (m, 2H), 2.387–2.406 (m, 4H), 6.454–6.478 (d, 1H, *J* = 9.6 Hz), 7.398–7.415 (m, 4H, Ar-H), 7.921–7.945 (d, 1H, *J* = 9.9 Hz). ^13^C-NMR (100 MHz, DMSO-*d_6_*): δ 20.352, 28.972, 35.846, 114.682, 116.070, 116.658, 116.886, 129.993, 130.084, 132.678, 141.085, 160.996, 163.125, 163.201, 163.201, 163.453, 164.277, 193.426. 

*1-(4'-Bromophenyl)-7,8-dihydro-2,5(1*H*,6*H*)-quinolinedione *(**5j**). Yield: 40%, HPLC purity: 99.2%, mp: 211.8–212.9 °C, MS *m/z*: 319 (M^+^). ^1^H-NMR (300 MHz, DMSO-*d_6_*): δ 2.004–2.088 (m, 2H), 2.426–2.547 (m, 4H), 6.560–6.592 (d, 1H, *J* = 9.6 Hz), 7.088–7.266 (m, 2H, Ar-H), 7.681–7.709 (m, 2H, Ar-H), 8.045–8.078 (d, 1H, *J* = 9.9 Hz). ^13^C-NMR (100 MHz, DMSO-*d_6_*): δ 21.525, 29.052, 36.508, 114.926, 119.090, 123.748, 129.666 (2C), 133.516 (2C), 136.499, 137.245, 156.823, 163.089, 194.000.

*1-(4'-Methoxyphenyl)-7,8-dihydro-2,5(1*H*,6*H*)-quinolinedione* (**5k**). Yield: 45%, HPLC purity: 99.2%, mp: 165.3–165.8 °C, MS *m/z*: 269 (M^+^). ^1^H-NMR (400 MHz, DMSO-*d_6_*): δ 1.884–1.947 (m, 2H), 2.396–2.428 (m, 4H), 6.432–6.456 (d, 1H, *J=* 9.6 Hz), 7.076–7.098 (m, 2H, Ar-H), 7.219–7.242 (m, 2H, Ar-H), 7.902–7.927 (d, 1H, *J=* 10.0 Hz).^13^C-NMR (100 MHz, DMSO-*d_6_*): δ 21.132, 28.784, 36.199, 55.714, 113.799, 115.012 (2C), 117.911, 29.438 (2C), 130.308, 136.625, 159.290, 159.549, 162.631, 194.146. 

*1-(2',4’-Dichlorophenyl)-7,8-dihydro-2,5(1*H*,6*H*)-quinolinedione* (**5l**). Yield: 35%, HPLC purity: 99.2%, mp: 200.5–201.7 °C, MS *m/z*: 307 (M^+^). ^1^H-NMR (400 MHz, DMSO-*d_6_*): δ 1.904–2.004 (m, 2H), 2.191–2.264 (m, 1H), 2.429–2.548 (m, 4H), 6.503–6.527 (d, 1H, *J* = 9.6 Hz), 6.956–6.975 (d, 1H, Ar-H), 7.669–7.696 (m, 1H, Ar-H), 7.965–7.971 (d, 1H, Ar-H), 7.956–7.980 (d, 1H, *J* = 9.6 Hz). ^13^C-NMR (100 MHz, DMSO-*d_6_*): δ 20.840, 27.508, 35.876, 113.820, 117.871, 128.963, 129.909, 131.671, 132.373, 134.044, 134.860, 137.042, 157.838, 161.133, 193.586.

*1-(2',4’-Dimethyphenyl)-7,8-dihydro-2,5(1*H*,6*H*)-quinolinedione* (**5m**). Yield: 45%, HPLC purity: 99.0%, mp: 178.5–179.0 °C, MS *m/z*: 267 (M^+^). ^1^H-NMR (300 MHz, DMSO-*d_6_*): δ 1.968–2.083 (m, 2H), 2.034 (s, 3H, CH_3_), 2.189–2.262 (m, 1H), 2.380 (s, 3H, CH_3_), 2.441–2.572 (m, 3H), 6.570–6.594 (d, 1H, *J* = 9.6 Hz), 6.956–6.975 (m, 1H, Ar-H), 7.151–7.260 (m, 2H, Ar-H), 8.044–8.068 (d, 1H, *J* = 9.6 Hz). ^13^C-NMR (100 MHz, DMSO-*d_6_*): δ 17.462, 21.381, 21.653, 28.432, 36.639, 114.867, 119.100, 127.321, 128.595, 132.503, 134.072, 134.633, 137.053, 139.880, 157.432, 162.943, 194.269. 

#### 3.1.5. General Procedure for the Preparation of **6**

*5-Hydroxy-1-phenyl-5,6,7,8-tetrahydroquinolin-2(1*H*)-one* (**6a**). To a solution of ethanol (20 mL) and **5a** (1.0 g) was added NaBH_4 _(0.02 mol). The mixture was stirred at room temperature for 5 h. Ethanolwas removed completely and a white solid was obtained. The white solid was dissolved in water (30 mL), and pH was adjusted to 4 with diluted HCl. Crystallization of a white solid occurred, which was filtered and recrystallized in methanol to afford a white solid. Yield: 87%, HPLC purity: 99.1%, mp: 218.5–220.0 °C, MS *m/z*: 241 (M^+^). ^1^H-NMR (400 MHz, DMSO-*d_6_*): δ 1.635–1.681 (m, 1H), 1.707–1.900 (m, 4H), 2.010–2.338 (m, 2H), 4.653 (s, 1H, OH), 6.5677–6.598 (m, 1H, *J=* 9.3 Hz), 7.129–7.262 (m, 2H, Ar-H), 7.446–7.521 (m, 3H, Ar-H), 7.545–7.607 (m, 1H).^13^C-NMR (100 MHz, DMSO-*d_6_*): δ21.4 72, 28.924, 36.470, 114.684, 119.004, 127.771 (2C), 129.449, 130.133 (2C), 136.950, 137.491, 157.133, 163.212, 194.063.

*5-Hydroxy-1-(4'-chlorophenyl)-5,6,7,8-tetrahydroquinolin-2(1*H*)-one* (**6d**). Yield: 82%, HPLC purity: 99.1%, mp: 160.9–161.7 °C, MS *m/z*: 275 (M^+^). ^1^H-NMR (400 MHz, DMSO-*d_6_*): δ 1.659–1.701 (m, 1H), 1.806–1.884 (m, 1H), 2.071–2.139 (m, 4H), 2.010–2.338 (m, 2H), 4.648 (s, 1H, OH), 6.565–6.589 (m, 1H, *J=* 9.6 Hz), 7.086–7.266 (m, 2H, Ar-H), 7.474–7.490 (m, 2H, Ar-H), 7.506–7.513 (m, 1H). ^13^C-NMR (100 MHz, DMSO-*d_6_*): δ 17.411, 28.694, 30.975, 66.243, 116.769, 118.043, 119.096 (2C), 122.231, 124.802, 134.834, 136.711, 139.595, 141.563 (2C), 144.996. 

*5-Hydroxy-1-(2'-ﬂuorophenyl)-5,6,7,8-tetrahydroquinolin-2(1*H*)-one* (**6h**). Yield: 87%, HPLC purity: 98.1%, mp: 186.9–188.8 °C, MS *m/z*: 259 (M−H)^+^. ^1^H-NMR (400 MHz, DMSO-*d_6_*): δ 1.732 (m, 6H), 4.439–4.450 (m, 1H), 5.187–5.203 (m, 1H), 6.395–6.418 (m, 1H, *J* = 9.2 Hz), 7.341–7.369 (m, 2H, Ar-H), 7.432–7.457m, 1H, *J* = 9.4 Hz), 7.537–7.560 (m, 2H, Ar-H). ^13^C-NMR (100 MHz, DMSO-*d_6_*): δ 17.493, 28.059, 30.935, 64.616, 116.622, 116.820, 117.576, 117.812, 125.868, 131.201, 142.796, 144.726, 156.154, 158.619, 161.701.

*5-Hydroxy-1-(4'-ﬂuorophenyl)-5,6,7,8-tetrahydroquinolin-2(1*H*)-one* (**6i**). Yield: 78%, HPLC purity: 99.1%, mp: 186.9–188.8 °C, MS *m/z*: 259 (M^+^).^1^H-NMR (400 MHz, DMSO-*d_6_*): δ 1.732 (m, 6H), 4.439–4.450 (m, 1H), 5.187–5.203 (m, 1H), 6.395–6.418 (m, 1H, *J* = 9.2 Hz), 7.341–7.369 (m, 2H, Ar-H), 7.432–7.457 (m, 1H, *J* = 9.4 Hz), 7.537–7.560 (m, 2H, Ar-H). ^13^C-NMR (100 MHz, DMSO-*d_6_*): δ 17.493, 28.059, 30.935, 64.616, 116.622, 116.820, 117.576, 117.812, 125.868, 131.201, 142.796, 144.726, 156.154, 158.619, 161.700.

*5-Hydroxy-1-(4'-bromophenyl)-5,6,7,8-tetrahydroquinolin-2(1*H*)-one* (**6j**). Yield: 91%, HPLC purity: 99.2%, mp: 179.5–180.6 °C, MS *m/z*: 319 (M^+^). ^1^H-NMR (400 MHz, DMSO-*d_6_*): δ 1.721–2.010 (m, 6H), 4.439–4.462 (m, 1H), 5.174–5.189 (m, 1H), 6.367–6.390 (m, 1H, *J=* 9.2 Hz), 7.177–7.198 (m, 2H, Ar-H), 7.511–7.535 (m, 1H, *J* = 9.4 Hz), 7.699–7.717 (m, 2H, Ar-H). ^13^C-NMR (100 MHz, DMSO-*d_6_*): δ 17.730, 28.624, 30.966, 64.761, 117.637, 121.825, 131.063 (2C), 132.742 (2C), 138.044, 142.354, 144.711, 162.151. 

*5-Hydroxy-1-(4'-methoxyphenyl)-5,6,7,8-tetrahydroquinolin-2(1*H*)-one *(**6k**). Yield: 75%, HPLC purity: 99.0%, mp: 181.5–181.9 °C, MS *m/z*: 271 (M^+^). ^1^H-NMR (400 MHz, DMSO-*d_6_*): δ 1.514–1.749 (m, 4H), 2.015 (m, 2H), 3.803 (S, 3H, OCH_3_), 4.431–4.442 (m, 1H), 5.142–5.157 (m, 2H), 6.367–6.343 (m, 1H, *J=* 9.2 Hz), 6.926–7.185 (m, 4H, Ar-H), 7.481–7.504 (m, 1H, *J* = 9.2 Hz).^13^C-NMR (100 MHz, DMSO-*d_6_*): δ 17.730, 28.677, 31.019, 55.653, 64.830, 114.829, 114.875, 117.377, 117.576, 129.621 (2C), 131.254, 142.041, 145.436, 159.114, 162.524.

*5-Hydroxy-1-(2',4’-dichlorophenyl)-5,6,7,8-tetrahydroquinolin-2(1*H*)-one* (**6l**). Yield: 91%, HPLC purity: 99.2%, mp: 210.9–217.4 °C, MS *m/z*: 309 (M^+^). ^1^H-NMR (400 MHz, DMSO-*d_6_*): δ 1.533–1.640 (m, 2H), 1.696–1.833 (m, 3H), 2.104–2.151 (m, 2H), 3.849–3.864 (m, 1H), 4.454–4.491 (m, 1 H), 6.396–6.420 (m, 1H, *J* = 9.3 Hz), 7.457–7.479 (m, 1H, Ar-H), 7.553–7.577 (m, 1H, *J* = 9.4 Hz), 7.594–7.621 (m, 1H, Ar-H), 7.890–7.896 (m, 2H, Ar-H). ^13^C-NMR (100 MHz, DMSO-*d_6_*): δ 17.233, 27.471, 30.614, 64.212, 117.285, 117.499, 128.591, 129.606, 131.856, 132.611, 134.046, 135.045, 142.376, 143.841, 161.006.

*5-Hydroxy-1-(2',4’-dimethyphenyl)-5,6,7,8-tetrahydroquinolin-2(1*H*)-one* (**6m**). Yield: 92%, HPLC purity: 99.1%, mp: 148.9–149.5 °C, MS *m/z*: 269 (M^+^). ^1^H-NMR (400 MHz, DMSO-*d_6_*): δ 1.724–1.739 (m, 5H), 1.865–1.904 (s, 3H, CH_3_), 1.986–2.089 (m, 1H), 2.322 (S, 3H, CH_3_), 4.435–4.446 (m, 1H), 5.119–5.160 (m, 1H), 6.354–6.377 (m, 1H, *J* = 9.3 Hz), 6.926–7.185 (m, 4H, Ar-H), 7.536–7.513 (m, 1H, *J* = 9.4 Hz). ^13^C-NMR (100 MHz, DMSO-*d_6_*): δ 17.524, 28.227, 30.295, 64.616, 116.622, 116.820, 117.576, 117.812, 125.868, 131.201, 142.796, 144.726, 156.154, 158.619, 161.624.

#### 3.1.6. *1-(2-Chloro-4-fluorophenyl)-5-hydroxy-2-oxo-1,2-dihydroquinoline-3-carboxylic acid* (**7o**)

To dried quinoline (20 mL) was added **3o** (5.0 g) and copper (0.1 g). The mixture was stirred and heated to 140–150 °C and refluxed for 8 h. When the suspension was cooled in the air to room temperature, compound **7o** was obtained, which upon column chromatography (EtOAc/petroleum ether) to yield a white solid. Yield: 34%, HPLC purity: 99.0%, mp: 181.5–181.9 °C, MS *m/z*: 334 (M^+^). ^1^H-NMR (400 MHz, DMSO-*d_6_*): δ 6.003–6.024 (d, 1H), 6.742–6.762 (d, 1H), 7.345–7.386 (d, 1 H), 7.462–7.480 (d, 1H), 7.664–686 (d, 1H), 7.709–7.751 (d, 1H), 7.812–7.824 (d, 1H), 9.154 (S, 1H), 11.073 (S, 1H).^13^C-NMR (100 MHz, DMSO-*d_6_*): δ 106.199, 107.946, 109.136, 117.993, 118.214, 119.731, 123.947, 130.023, 131.023, 131.374, 134.143, 134.662, 134.700, 138.957, 142.794, 156.007, 156.243, 158.479, 161.408, 163.918.

#### 3.1.7. General Procedure for the Preparation of **8i** and **8k**

*5-Hydroxyimimo-1-(4-methoxyphenyl)-5,6,7,8-tetrahydroquinolin-2(1*H*)-one* (**8i**). To **5i** (1 g), hydroxylammonium chloride (280 mg), anhydrous sodium acetate (330 mg), and water-ethanol (1:1, 50 mL) were added, the mixture was stirred and heated to reflux for 4 h. The white solid which formed was filtered off. Yield: 82%, HPLC: 99.4%; mp: 112.9–113.2 °C, MS *m/z*: 284 (M^+^). ^1^H-NMR (500 MHz, DMSO-*d_6_*): δ 1.638–1.688 (m, 2H), 2.177–2.202(m, 2H), 2.495–2.538(m, 2H), 3.813 (m, 3H), 6.393–6.413 (m, 1H, *J* = 10 Hz), 7.047–7.065 (m, 2H, Ar-H), 7.161–7.178 (m, 2H, Ar-H), 7.942–7.961 (m, 1H, *J* = 10*Hz*), 10.887 (m, 1H, OH). ^13^C-NMR (125 MHz, DMSO-*d_6_*): δ 20.268, 21.729, 28.288, 55.864, 110.832, 115.058 (2C), 118.561, 129.792, 131.020, 136.087 (2C), 148.473, 151.206, 159.490, 162.524.

*5-Hydroxyimimo-1-(4-fluorophenyl)-5,6,7,8-tetrahydroquinolin-2(1*H*)-one* (**8k**). Yield: 82%, HPLC: 97.0%; mp: 121.9–122.2 °C, MS *m/z*: 272 (M^+^). ^1^H-NMR (500 MHz DMSO-*d_6_*): δ 1.663–1.688 (m, 2H), 2.166–2.190(m, 2H), 2.499–2.544 (m, 2H), 6.415–6.435 (m, 1H, *J* = 10 Hz), 7.336–7.386 (m, 4H, Ar-H), 7.962–7.981 (m, 1H, *J* = 10 Hz), 10.914 (m, 1H, OH). ^13^C-NMR (125 MHz DMSO-*d_6_*): δ 20.237, 21.692, 28.260, 111.004, 116.689, 116.871, 118.604, 120.983, 131.053, 136.660, 148.050, 151.113, 161.195, 162.362, 163.146.

#### 3.1.8. *5-Amino-1-phenyl-5,6,7,8-tetrahydroquinolin-2(1*H*)-one* (**9a**)

To the suspension of **5a** (478 mg) and Al-Ni (1:1, 340 mg) in ethanol (30 mL) was quickly added NaOH (10%, 6 mL) and kept stirring at 50 °C. After the disappearance of starting materials, the solution was cooled, filtered, and extracted with dichloromethane. After removal of the solvent, recrystallization from EtOAc, slight white crystals were obtained. Yield: 35%, HPLC purity: 99.0%; mp: 186.5–187.3 °C. MS *m/z*: 241 (M^+^). ^1^H-NMR (300 MHz, DMSO-*d_6_*): δ 1.597–1.676 (m, 2H), 1.753–1.907 (m, 2H), 2.044–2.170 (m, 2H), 3.849–3.864 (m, 1H), 6.563–6.594 (m, 1H, *J* = 9.3*Hz*), 7.143–7.265 (m, 2H, Ar-H), 7.413–7.484 (m, 3H, Ar-H), 7.501–7.532 (m, 1H, *J* = 9.3 Hz). ^13^C-NMR (100 MHz, DMSO-*d_6_*): δ 18.239, 29.010, 32.367, 47.815, 118.330, 119.152, 128.211, 128.918, 129.982, 130.011, 138.688, 141.438, 144.507, 163.522.

### 3.2. Biological Assay

NIH3T3 cells were grown in RPMI1640 medium with 10% fetal bovine serum at 37 °C in humidified incubator with 5% CO_2_. Cell proliferation was monitored by the 3-[4,5-dimethyl-2-thiazolyl]-2,5-diphenyl-2*H*-tetrazolium bromide (MTT) assay. More specifically, NIH3T3 cells were plated in a 96-well plate at 10,000 cells per well, cultured for 4 h in complete growth medium, and then treated with gradient concentrations of the compounds for 72 h. After that, 0.5% MTT solution was added to each well and incubated for another 4 h, the formazan formed was extracted by adding DMSO and mixing for 15 min. The optical density was recorded at 490 nm with a Multiskan Ascent 354 microplate reader (Thermo Labsystems, Helsinki, Finland). The absorption was processed by Ascent Software™ and IC_50_ values were obtained from the dose-response curves.

## 4. Conclusions

In summary, our studies have shown that N1-substituted phenylhydroquinolinones are a promising scaffold for the discovery of novel PFD analogs, which can maintain the structural requirements for multiple target inhibition. Most compounds prepared possessed potent anti-fibrosis activity and the most active compound, **7o**, exhibited good inhibition against NIH3T3 cell proliferation. This interesting feature may also guide the design and discovery of new multiple-target anti-fibrosis agents
